# Spray mist reduction by means of a high-volume evacuation system—Results of an experimental study

**DOI:** 10.1371/journal.pone.0257137

**Published:** 2021-09-03

**Authors:** Martin Koch, Christian Graetz

**Affiliations:** 1 Technical Academy DÜRR DENTAL SE, Bietigheim-Bissingen, Germany; 2 Clinic of Conservative Dentistry and Periodontology, University of Kiel, Kiel, Germany; Thamar University, Faculty of Dentistry, YEMEN

## Abstract

**Objectives:**

High-speed tooth preparation requires effective cooling to avoid thermal damage, which generates spray mist, which is a mixture of an aerosol, droplets and particles of different sizes. The aim of this experimental study was to analyze the efficacy of spray mist reduction with an intraoral high-volume evacuation system (HVE) during simulated high-speed tooth preparation for suboptimal versus optimal suction positions of 16 mm sized cannulas and different flow rates of the HVE.

**Material and methods:**

In a manikin head, the upper first premolar was prepared with a dental turbine, and generated particles of 5–50 microns were analyzed fifty millimeters above the mouth opening with the shadow imaging technique (frame: 6.6×5.3×1.1 mm). This setup was chosen to generate a reproducible spray mist in a vertical direction towards an imaginary operator head (worst case scenario). The flow rate (FR) of the HVE was categorized into five levels (≤120 l/min up to 330 l/min). The number of particles per second (NP; p/s) was counted, and the mass volume flow of particles per second (MVF; μg/s*cm^3^) was calculated for 10 sec. Statistical tests were nonparametric and two-sided (p≤0.05).

**Results:**

With increasing flow rate, the NP/MVF values decreased significantly (eta: 0.671/0.678; p≤0.001). Using a suboptimally positioned cannula with an FR≤160 l/min, significantly higher NP values (mean±SD) of 731.67±54.24 p/s (p≤0.019) and an MVF of 3.72±0.42 μg/s*cm^3^ (p≤0.010) were measured compared to those of the optimal cannula position and FR≥300 l/min (NP/MVF: 0/0). No significant difference in NP and MVF was measurable between FR≥250 l/min and FR>300 l/min (p = 0.652, p = 0.664).

**Conclusion:**

Within the limitations of the current experimental study, intraoral high-flow rate suction with ≥300 l/min with an HVE effectively reduced 5–50 μm sized particles of the spray mist induced by high-speed tooth preparation with a dental turbine.

## Introduction

Before the pandemic of SARS-CoV-2, droplets and aerosols were already indicated as possible risks for infections in dentistry [1], by adequate protective measures for dental staff against pathogens transmitted via droplets, splatter or aerosols from the patients’ oral cavity were of only small scientific interest [2, 3]. However, this changed rapidly during the first year of the pandemic of SARS-CoV-2 in 2020 due to the disruption in health care and dentistry worldwide [4]. Causes for risks are (a) close contact between the patient’s mouth and dental professionals and (b) the formation of spray mist during dental interventions. Spray mist is an inhomogeneous mixture of air, water and solids that is produced by various water-cooled instruments in dental practice. It contains different particle sizes classified as aerosol, splatter or droplets [5] with a possible risk for pathogen transmission. Particles of different sizes, especially those under 50 μm, are not visible to the naked eye and can be suspended in the air for different times depending on the air temperature, humidity and turbulence [6].

With the advent of SARS-CoV-2 infection, questions concerning the potential for the spread of infections from this spray mist may arise. Regarding the exact infection dose required in virus copies to trigger an infection, e.g., with SARS-CoV-2, which is currently unknown, dental professionals must be protected from any infection through spray mist. An early investigation in the seventies indicated that intraoral suction systems with a high flow rate (FR) were favored in dentistry–the so-called high-volume evacuation system (HVE) [7]. According to ISO 10637:2018, a suction system with an FR>250 l/min can be classified as an HVE and is highly recommended as one of the spray mist reducing procedures in dentistry with high efficacy [8]. However, dentists often tend to choose devices that offer the greatest convenience and comfort, e.g., suction systems optimized for high vacuum in combination with saliva ejectors with small diameter instead of systems with high-flow rates and appropriately sized cannula (e.g., ≥10 mm) with noise inherent in the system [9]. Therefore, it is not surprising that the results of a recently published intervention review found that for all kinds of suction methods, the evidence is of very low certainty due to heterogeneity, risk of bias, small sample sizes and wide confidence intervals comparing different methods of dental suction [10]. This experimental study analyzed the efficacy of spray mist reduction among high-flow evacuations with 16 mm sized suction cannulas during simulated high-speed tooth preparation with a turbine in a manikin head. Particle sizes of 5–50 microns were measured in different settings for (1) two intraoral suction positions of the cannulas (optimal vs. suboptimal) and (2) five levels of the flow rate (120 l/min ≤ x ≤ 330 l/min) of the intraoral HVE.

## Material and methods

### Experimental setup—manikin head and test tooth

**Fig 1:**
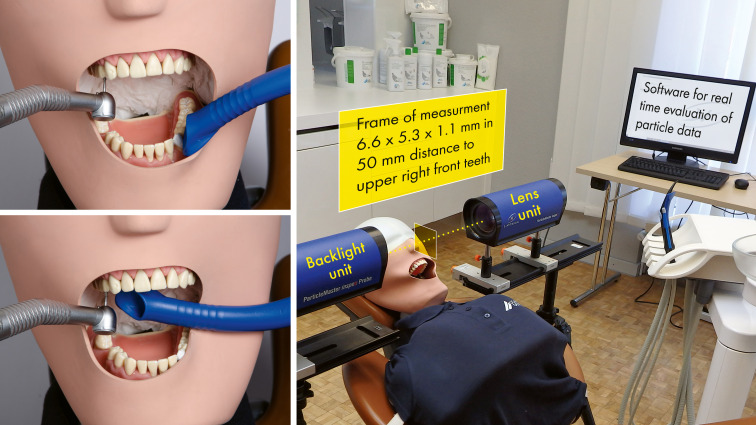
Overview of the experimental setup. Shown on the right side is the overview of the experimental setup with the imaging process technique (ParticleMaster, Lavision, Göttingen, Germany). As shown on left side, a dental turbine (Super-Torque LUX 3 650 B, Kavo, Biberach, Germany) was used for tooth preparation of the first right upper premolar, and a 16 mm sized suction cannula (Universal Cannula, DÜRR DENTAL SE, Bietigheim-Bissingen, Germany) was utilized in a suboptimal position in the opposite jaw (upper left side) and an optimal position (approximately 10 mm from the tooth (lower left side). The experimental setup was the same for all three types of cannulas. Dental turbines and cannulas were held in place by tripods during the tests.

A setup was chosen to generate a reproducible spray mist in a vertical direction towards an imaginary operator head sitting at a twelve o’clock position behind the manikin’s head in the lying position (Frasaco, Tettnach, Germany) during high-speed tooth preparation for ten seconds with a dental turbine at 400000 rpm (Super-Torque LUX 3 650 B, Kavo, Biberach, Germany) of an upper right first premolar (Fig 1). No removal of any tooth substance was done. The dental turbine and suction cannula were fixed by tripods during the tests. The volume of cooling water for tooth preparation was set to 58 ml/min, which is in line with the manufacturer’s specifications.

To simulate different settings for suction, 16 mm suction cannulas (selected for each test: Universal Cannula, Universal Cannula Petito, Aerosol Cannula; DÜRR DENTAL SE, Bietigheim-Bissingen, Germany) were utilized in (1) optimal versus suboptimal positions to the test tooth and with (2) five different categories of flow rate (l/min) of the HVE (Variosuc, DÜRR DENTAL SE, Bietigheim-Bissingen, Germany). A suboptimal cannula position for preparation of an upper right premolar was defined as the buccal side of the left lower first premolar by a mouth opening of approximately 50 mm (Fig 1). We randomized the start frequency for the type of cannula in each category of flow rate via https://www.random.org.

The flow rate was controlled by means of a slide on the suction handpiece and categorized into five levels of flow rate (FR-1: ≤120 l/min; FR-2: >120≤160 l/min; FR-3: >160≤250 l/min; FR-4: >250≤300 l/min; FR-5: >300≤330 l/min). The flow rate (l/min) was measured at the cannula tip using a calibrated float volume flowmeter (ROTA G 4.4000 SW = N4 10×, Rota Yokogawa, Wehr, Germany). All trials for optimal and suboptimal cannula positions in each of the five categories of flow rates were repeated with all three suction cannulas twice (n = 60).

Overall, with this experimental setup, a worst-case scenario was simulated (e.g., insufficient working distance between dentist and patient, insufficient suction position of the cannula, low flow rate of the HVE) to control for different factors influencing particle reduction.

### Particle evaluation

The particle emission was measured by the shadow imaging technique (ParticleMaster, Lavision, Göttingen, Germany) with pulsed background lighting (image frequency: 12.95 Hz; pulse duration of the light source: 0.4 μs; shooting method: double frame mode with an exposure time of 42 μs; interval between two images: 10 μs). Each measurement involved analyzing 127 single images (measurement time: 10 s) using the DaVis software solution (Version 10.1.1.60438, Lavision, Göttingen, Germany) in a frame measuring 6.6×5.3×1.1 mm fifty millimeters above the upper right front teeth. Particles between 5 μm (resolution limit) and 50 μm (cut off) could be analyzed with the described setup.

### Outcomes

The number of particles NP [p/s], between 5 μm and 50 μm, was counted as they passed through the measurement frame. The mass volume flow of the particles per second MVF [μg/s*cm^3^] was calculated.

### Statistical analysis

Data acquisition and collection were performed with Microsoft Excel (Microsoft Excel 16, Microsoft Corporation, One Microsoft Way Redmond, WA, USA). Tables were created and entered into SPSS Statistics (SPSS Statistics 24, IBM, Chicago, IL, USA) for statistical analysis. No initial power calculation for the study was performed, as this experimental study was designed to develop a standardized study protocol for further research questions. The presence of a normal distribution was tested by Kolmogorov-Smirnov and Shapiro-Wilk tests. The distribution was not normal. Subsequently, a mean value comparison was performed using the Kruskal-Wallis test to detect significant differences according to NP and MVP between the five categories of flow rate. For the difference between the optimal and suboptimal positions of the cannula, the Mann-Whitney U-test was used. To describe the possible correlation between categories of flow rate and the NP/MVP values, the eta-coefficient was used. All tests were two-sided; statistical significance was assumed if p≤0.05.

## Results

### Flow rate FR

**Fig 2:**
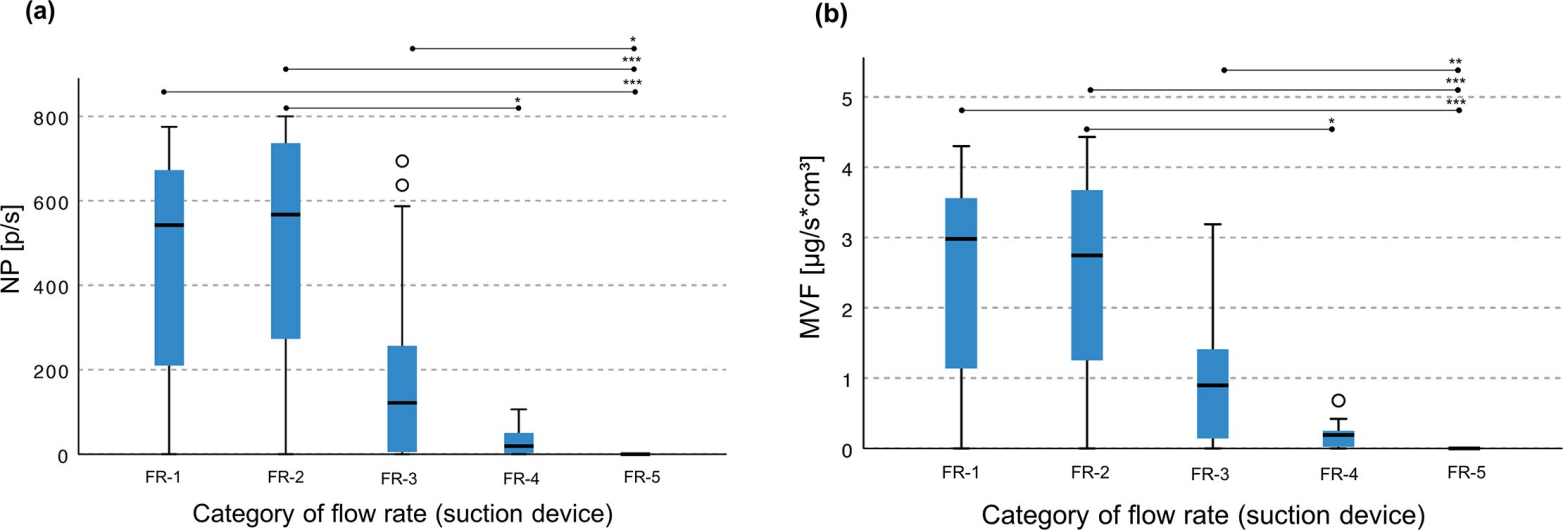
Comparison of all 16 mm sized cannulas independent of their intraoral position for (a) the number of counted particles/second [NP] and (b) the mass volume flow of the particles/second [MVF] per category of flow rate [FR]. Distribution of the results (mean±SD) for the (a) number of counted particles per second (p/s) and (b) mass volume flow of the particles per second (μg/s*cm^3^) according to the five categories of flow rate (FR-1: ≤120 l/min; FR-2: >120≤160 l/min; FR-3: >160≤250 l/min; FR-4: >250≤300 l/min; FR-5: >300≤330 l/min) of the high-volume evacuation system (HVE) for all three cannulas (Universal Cannula, Universal Cannula Petito, Aerosol Cannula) together and independent of their position (optimal and suboptimal). Significant differences between the FR categories are shown in the upper area (Kruskal-Wallis test with *p≤0.05; **p≤0.01; ***p≤0.001).

Due to software errors during recording in three test trials, only 57 test trials of 60 (95%) could be analyzed for NP and MVF. Without differentiation of the cannula position, the NP and MVF values of categories FR-1 to FR-3 were always significantly higher than those of FR-5 (p≤0.05; Fig 2). Only in the category FR-5 of the highest flow rate of ≥300 l/min were zero particles detectable for NP and MVF. No significant difference could be detected between FR-4 and FR-5, whether for NP or MVF.

We found a negative correlation between flow rate and NP (effect size eta = 0.671; p≤0.001) as well as MVF (effect size eta = 0.678; p≤0.001).

### Intraoral suction position of the cannula

After separation for optimal versus suboptimal position of the cannulas, we measured in categories of FR-1 up to FR-4 always significant lower values for the optimal position compared to the suboptimal position of the cannula of NP (98.06±184.11 vs. 317.73±312.11; p = 0.008) and MVF (0.47±0.86 vs. 1.68±1.60; p = 0.007). In the category with the highest flow rate FR-5 of the intraoral HVE, no particles between 5–50 μm generated by the simulated high-speed tooth preparation were detectable for either cannula position (Table 1).

**Table 1:** Comparison of the optimal versus suboptimal intraoral suction position of the 16 mm cannulas.

	position of the cannula	FR-1 (mean±SD)	FR-2 (mean±SD)	FR-3 (mean±SD)	FR-4 (mean±SD)	FR-5 (mean±SD)
**Number of counted particles per second (NP)**	suboptimal	599.00± 129.04	731.67± 54.24	321.22± 248.12	39.44± 37.61	0
	optimal	233.12± 252.37	254.33± 233.81	7.18±11.79	0	0
**P-value between the optimal and suboptimal positions** *		*0*.*01*	*0*.*002*	*0*.*001*	*0*.*036*	*n*.*a*.
**Mass volume flow of the particles per second (MVP)**	suboptimal	3.28±0.66	3.72±0.42	1.66±1.00	0.23±0.21	0
	optimal	0.01±0.01	1.19±1.11	0.80±0.10	0	0
**P-value between the optimal and suboptimal positions** *		*0*.*009*	*0*.*002*	*0*.*004*	*0*.*035*	*n*.*a*.

The results (mean±SD) for the number of counted particles per second (NP; p/s) and mass volume flow of the particles per second (MVF; μg/s*cm^3^) according to the five categories of flow rate (FR-1: ≤120 l/min; FR-2: >120≤160 l/min; FR-3: >160≤250 l/min; FR-4: >250≤300 l/min; FR-5: >300≤330 l/min).

FR: flow rate (l/min); NP: number of particles counted per second (p/s); MVF: mass volume flow of the particles per second (μg/s*cm^3^)

*Mann-Whitney U-test

## Discussion

It is indisputable that there is a need for effective cooling of the work areas to avoid thermal damage, which must be carried out for high-speed tooth preparation (approx. 50 ml/min) [11]. From a clinical point of view, a suction system can be considered sufficient if, in addition to liquids, solid particles (tooth parts, calculus) also completely eliminate the spray mist without affecting the cooling of the tooth. However, this goal can be reduced intentionally or unintentionally by various situations.

We found under standardized experimental conditions that, with a higher level of flow rate at the cannula, significantly lower values for NP and MVF occurred. In particular, intraoral evacuation generates a counterflow, which in turn slows down the emitted particles generated by high-speed preparation. Ideally, this intraoral suction is so strong that no particles leave the mouth region. We found in the current study that a flow rate of at least 300 l/min (Table 1, Fig 2) caused more noise and intraoral cooling of the air, which could lead to discomfort [9]. Additionally, it has to be assumed that with an excessively small distance between the dental turbine and suction cannula (<10 mm), the cooling spray is directly eliminated with such high flow rates. This must be avoided at all costs to reduce potential thermal hazards to the pulp and supporting tissues [12].

It has long been known that a powerful intraoral HVE with a high flow rate and appropriately sized tubes/cannulas successfully reduces spray mist [7]. Hence, if professionals use only a saliva extractor or a cannula with a small diameter leading to a low flow rate, the number of particles leaving the patient’s mouth opening dramatically increase. In the present simulated treatment situation, a lower flow rate of 160 l/min was not enough to prevent particle emission immediately above the mouth opening. Irrespective of simple methods for the control of spray mist already available [13], dentists did not apply those methods mostly before the pandemic of SARS-CoV-2 because of low awareness of health risks, working habits and economic factors [14].

**Fig 3:**
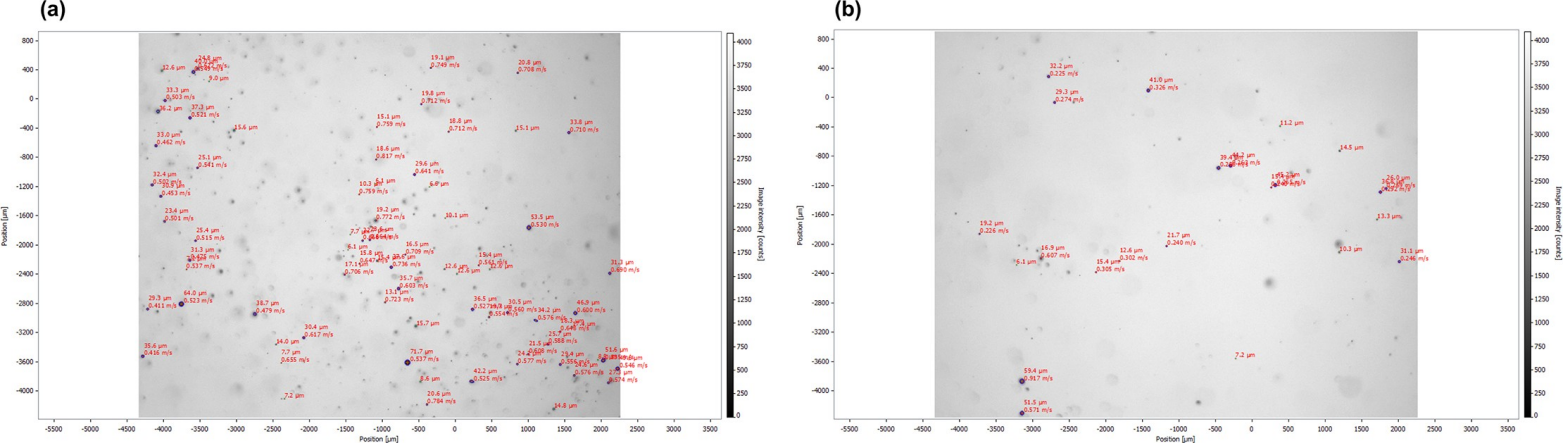
Example illustration of the distribution of the particles in a spray mist cloud by low flow rate (FR-2) of the HVE for (a) suboptimal versus (b) optimal positioned 16 mm sized cannula. Of the distribution of the particles in a spray mist cloud caused by a dental turbine and an intraoral high-volume evacuation system (HVE) set to a low flow rate of FR-2 >120≤160 l/min with the imaging process technique (ParticleMaster, Lavision, Göttingen, Germany); (a) suboptimal positioned 16 mm sized cannula in the opposite jaw versus (b) optimal positioned 16 mm sized cannula near tooth preparation. The imaging software determined the number and size of particles as well as the velocity of each individual particle between 5 μm and 50 μm.

Therefore, the flow rate at the suction cannula is the crucial physical parameter for reducing spray mist. It is surprising that many intraoral HVE are optimized for vacuum and not for flow rate. Many parameters influence the flow rate, some of which are more complicated to control (e.g., the suction unit, line diameter and layout, distance between suction unit and the user), while other parameters are still easy to check, such as the diameter of the cannula (including blocking with foreign objects) and the intraoral position. As proven before, with larger cannula diameters, splatter contamination in the dental operatory is significantly reduced [15]. Additionally, the intraoral position of the cannula and operator’s position significantly influence the results of spray mist reduction [15, 16]. We found that in addition to the highest category FR-5 (≥300 l/min), all lower categories of flow rate displayed a significant difference in NP and MVF (p≤0.036) between a suboptimal position of the cannula (distance: 50 mm) and an optimal utilized cannula (approximately 10 mm near the tooth). An example illustration of both simulated situations is given in Fig 3 for a low flow rate of FR-2 with >120≤160 l/min. Therefore, the intraoral position of the cannula should be considered especially for treatments with high-speed turbines that generate more spray mist [16] or if treatments are carried out without assistance while avoiding suctioning of the patient’s intraoral soft tissues [17].

In vitro investigations have shown high efficacy of spray mist reduction during dental treatments [18, 19], leading to minimization of contaminated area to such an extent that not even the treatment team comes in contact with the spray mist [3]. Nevertheless, the results of the studies that measured spray mist and droplets via indirect techniques such as coloring the cooling water [3, 15] or counting droplets on filter paper [20, 21] as well as a combination of both systems [22] have not alone been suitable to provide evidence-based recommendations for safety against aerogenic transmission of infections. However, investigations measuring small particles under five microns directly could fail to indicate differences between various suction devices due their inherent measuring technique [16].

Different limitations of the current study can be pointed out. Our in vitro results cannot be transferred 1:1 to a clinical setting according to the experimental nature of the data, including a low number of samples, and do not permit a detailed analysis of which factors may have the most deleterious influence on the efficacy of spray mist reduction. For instance, as other experimental studies tested for various spray mist generating procedures [21, 23], our simulation included only one high-speed tooth preparation with a dental turbine without destruction of the test tooth and no breathing of the manikin. It must be assumed that additional particles as a result of tooth preparation and breathing airflow will further increase the total number of countable particles. Additionally, minimal changes in the room conditions (e.g., air ventilation, temperature) can have an unknown effect on the direction of the particle [6]. This must be noted, as our measuring method used a small frame of 6.6×5.3×1.1 mm for particle counting. The restriction of the particle resolution of 5–50 microns also has to be mentioned as a limitation. The infectivity of virus-laden particles correlates with their sizes, and smaller particles are potentially more infectious than larger particles; e.g., the coronavirus RNA genome was detected in 40% of aerosol particles smaller than 5 μm and in only 30% of larger particles (>5 μm) [24]. However, for particles larger than 50 μm, it can be assumed that they fall relatively rapidly [15] and have to be managed with adequate surface disinfection after treatment [25]. Furthermore, due to the border lines of measurable particles in our investigation, the suction efficacy of an intraoral HVE is overestimated when directly compared with other particle measurement methods [16]. Therefore, to control all particles, additional protective measures (e.g., use of eye protection, masks, gloves, clothing coverage, and preprocedural antiseptic mouthwash) are strongly recommended in dentistry [25, 26]. As we chose a case scenario with reproducible spray mist in a vertical direction towards an imaginary operator head (worst case scenario) and a fixed particle measuring frame, we were unable to present data to detect differences in the direction of spray mist. Previous investigations found that scenarios where the spray mist is frequently directed towards the air of the operatory are the most difficult to control [3, 15, 16].

Overall, experimental research data are necessary to generate a hypothesis or develop methods for clinical studies, which are required promptly, as the influence of airborne microorganism transmission is still unclear. Therefore, every effort should be made to further improve the knowledge to develop procedures and safety protocols in dentistry [27].

## Conclusions

Within the limits of the in vitro study design and the chosen experimental setup, intraoral high-volume suction had a beneficial effect on reducing particles sized 5–50 microns induced by the spray mist of high-speed tooth preparation, and this effect was improved with higher flow rates.

## References

[1] Walmsley AD. Potential hazards of the dental ultrasonic descaler. Ultrasound Med Biol. 1988;14(1):15–20. Epub 1988/01/01. doi: 10.1016/0301-5629(88)90159-7 . 3279684

[2] Bentley CD, Burkhart NW, Crawford JJ. Evaluating spatter and aerosol contamination during dental procedures. J Am Dent Assoc. 1994;125(5):579–84. Epub 1994/05/01. doi: 10.14219/jada.archive.1994.0093 . 8195499

[3] Reitemeier B, Jatzwauk L, Jesinghaus S, Reitemeier C, Neumann K. [Effective reduction of aerosol—possibilities and limitations]. ZMK. 2010;26:662–73 [Article in German].

[4] Benzian H, Niederman R. A Dental Response to the COVID-19 Pandemic-Safer Aerosol-Free Emergent (SAFER) Dentistry. Front Med (Lausanne). 2020;7:520. Epub 2020/09/10. doi: 10.3389/fmed.2020.00520; PubMed Central PMCID: PMC7434942. 32903453 PMC7434942

[5] Shiu EYC, Leung NHL, Cowling BJ. Controversy around airborne versus droplet transmission of respiratory viruses: implication for infection prevention. Curr Opin Infect Dis. 2019;32(4):372–9. Epub 2019/07/02. doi: 10.1097/QCO.0000000000000563 . 31259864

[6] Polednik B. Exposure of staff to aerosols and bioaerosols in a dental office. Building and Environment. 2021;187:1–13.

[7] Davies MH, Rosen M, Eccles JD, Marshal RJ. Criteria of air flow and negative pressure for high volume dental suction. Br Dent J. 1971;130(11):483–7. Epub 1971/06/01. doi: 10.1038/sj.bdj.4802680 . 4931933

[8] Samaranayake LP, Fakhruddin KS, Buranawat B, Panduwawala C. The efficacy of bio-aerosol reducing procedures used in dentistry: a systematic review. Acta Odontol Scand. 2021;79(1):69–80. Epub 2020/12/15. doi: 10.1080/00016357.2020.1839673 . 33307917

[9] Comisi JC, Ravenel TD, Kelly A, Teich ST, Renne W. Aerosol and spatter mitigation in dentistry: Analysis of the effectiveness of 13 setups. J Esthet Restor Dent. 2021. Epub 2021/02/02. doi: 10.1111/jerd.12717; PubMed Central PMCID: PMC8014276. 33522677 PMC8014276

[10] Kumbargere Nagraj S, Eachempati P, Paisi M, Nasser M, Sivaramakrishnan G, Verbeek JH. Interventions to reduce contaminated aerosols produced during dental procedures for preventing infectious diseases. Cochrane Database Syst Rev. 2020;10:CD013686. Epub 2020/10/14. doi: 10.1002/14651858.CD013686.pub2. 33047816 PMC8164845

[11] von Fraunhofer JA, Siegel SC, Feldman S. Handpiece coolant flow rates and dental cutting. Operative dentistry. 2000;25(6):544–8. Epub 2001/02/24. . 11203868

[12] Kwon SJ, Park YJ, Jun SH, Ahn JS, Lee IB, Cho BH, et al. Thermal irritation of teeth during dental treatment procedures. Restor Dent Endod. 2013;38(3):105–12. Epub 2013/09/07. doi: 10.5395/rde.2013.38.3.105 ; PubMed Central PMCID: PMC3761117. 24010075 PMC3761117

[13] Harrel SK. Airborne spread of disease—the implications for dentistry. J Calif Dent Assoc. 2004;32(11):901–6. Epub 2005/01/18. . 15651466

[14] Szymanska J. Dental bioaerosol as an occupational hazard in a dentist’s workplace. Ann Agric Environ Med. 2007;14(2):203–7. Epub 2008/02/06. . 18247451

[15] Graetz C, Bielfeldt J, Tillner A, Plaumann A, Dörfer C. Spatter contamination in dental practices–how can it be prevented? Rev Med Chir Soc Med Nat, Iaşi. 2014;118(4):1122–34. 25581979

[16] Kun-Szabo F, Gheorghita D, Ajtai T, Hodovany S, Bozoki Z, Braunitzer G, et al. Aerosol generation and control in the dental operatory: An in vitro spectrometric study of typical clinical setups. PLoS One. 2021;16(2):e0246543. Epub 2021/02/05. doi: 10.1371/journal.pone.0246543; PubMed Central PMCID: PMC7861533 our adherence to PLOS ONE policies on sharing data and materials, as Gabor Braunitzer did not participate in this study as an agent of dicomLAB Dental, Ltd. and dicomLAB Dental, Ltd. did not support this study in any way. The rest of the authors authors report no competing interests. 33539439 PMC7861533

[17] Mamoun JS. Clinical techniques of performing suctioning tasks and of positioning the high volume evacuation (HVE) attachment and inlet when assisting a dentist. A guide for dental assistants: part 2. Dent Assist. 2011;80(6):6, 8, 10–2. Epub 2012/03/01. . 22359818

[18] Harrel SK, Barnes JB, Rivera-Hidalgo F. Aerosol reduction during air polishing. Quintessence Int. 1999;30(9):623–8. Epub 2000/04/15. . 10765868

[19] Jacks ME. A laboratory comparison of evacuation devices on aerosol reduction. J Dent Hyg. 2002;76(3):202–6. Epub 2002/09/26. . 12271865

[20] Veena HR, Mahantesha S, Joseph PA, Patil SR, Patil SH. Dissemination of aerosol and splatter during ultrasonic scaling: A pilot study. J Infect Public Health. 2015. Epub 2015/01/08. doi: 10.1016/j.jiph.2014.11.004. 25564419

[21] Kaufmann M, Solderer A, Gubler A, Wegehaupt FJ, Attin T, Schmidlin PR. Quantitative measurements of aerosols from air-polishing and ultrasonic devices: (How) can we protect ourselves? PLoS One. 2020;15(12):e0244020. Epub 2020/12/16. doi: 10.1371/journal.pone.0244020; PubMed Central PMCID: PMC7737972. 33320905 PMC7737972

[22] Allison JR, Currie CC, Edwards DC, Bowes C, Coulter J, Pickering K, et al. Evaluating aerosol and splatter following dental procedures: Addressing new challenges for oral health care and rehabilitation. J Oral Rehabil. 2021;48(1):61–72. Epub 2020/09/24. doi: 10.1111/joor.13098 ; PubMed Central PMCID: PMC7537197. 32966633 PMC7537197

[23] Gross KB, Overman PR, Cobb C, Brockmann S. Aerosol generation by two ultrasonic scalers and one sonic scaler. A comparative study. J Dent Hyg. 1992;66(7):314–8. Epub 1992/09/01. . 1291635

[24] Leung NHL, Chu DKW, Shiu EYC, Chan KH, McDevitt JJ, Hau BJP, et al. Respiratory virus shedding in exhaled breath and efficacy of face masks. Nat Med. 2020;26(5):676–80. Epub 2020/05/07. doi: 10.1038/s41591-020-0843-2 . 32371934 PMC8238571

[25] Müller LK, Heider J, Frankenberger R, Graetz C, Jatzwauk L, Nagaba J, et al. German Guidelines: Dealing with aerosol-borne pathogens in dental practices. Dtsch Zahnärztl Z INT. 2020;2(6):164–9.

[26] ADA. Infection control recommendations for the dental office and the dental laboratory. ADA Council on Scientific Affairs and ADA Council on Dental Practice. J Am Dent Assoc. 1996;127(5):672–80. Epub 1996/05/01. doi: 10.14219/jada.archive.1996.0280 . 8642147

[27] Bizzoca ME, Campisi G, Lo Muzio L. An innovative risk-scoring system of dental procedures and safety protocols in the COVID-19 era. BMC Oral Health. 2020;20(1):301. Epub 2020/11/06. doi: 10.1186/s12903-020-01301-5; PubMed Central PMCID: PMC7609832. 33148254 PMC7609832

